# EmsB Microsatellite Analysis of *Echinococcus multilocularis* Specimens Isolated from Belgian Patients with Alveolar Echinococcosis and from Animal Hosts

**DOI:** 10.3390/pathogens14060584

**Published:** 2025-06-12

**Authors:** Sabrina Egrek, Jenny Knapp, Rosalie Sacheli, Khalid El Moussaoui, Philippe Léonard, Eva Larranaga Lapique, Laurence Millon, Sara Engelskirchen, Olivier Detry, Annick Linden, Marie-Pierre Hayette

**Affiliations:** 1 Department of Clinical Microbiology—Belgian National Reference Laboratory for Echinococcosis, Center for Interdisciplinary Research on Medicines (CIRM), University Hospital of Liege, 4000 Liege, Belgium; r.sacheli@chuliege.be (R.S.); kelmoussaoui@chuliege.be (K.E.M.); mphayette@chuliege.be (M.-P.H.); 2 EchinoLiège, University Hospital of Liege (CHU-ULiege), Avenue de l’Hôpital, 1, 4000 Liège, Belgium; philippe.leonard@chuliege.be (P.L.); olivier.detry@chuliege.be (O.D.); a.linden@uliege.be (A.L.); 3 Laboratory Chrono-Environment UMR CNRS 6249, University Marie & Louis Pasteur, 25030 Besançon, Francelmillon@chu-besancon.fr (L.M.); 4 Department of Parasitology-Mycology, National Reference Center for Echinococcoses, University Hospital of Besançon, 25030 Besançon, France; 5 Department of Infectious and Tropical Diseases, University Hospital of Liege, 4000 Liege, Belgium; 6 Department Clinic of Infectious Disease, University Hospital of Brussels, 1070 Brussels, Belgium; eva.larranagalapique@hubruxelles.be; 7 Fundamental and Applied Research for Animals and Health (FARAH), Faculty of Veterinary Medicine, University of Liege, 4000 Liege, Belgium; sara.engelskirchen@uliege.be; 8 Department of Abdominal Surgery and Transplantation, University Hospital of Liege, 4000 Liege, Belgium

**Keywords:** *Echinococcus multilocularis*, alveolar echinococcosis, human, animal hosts, Belgium, EmsB Microsatellite

## Abstract

Alveolar echinococcosis (AE), caused by *Echinococcus multilocularis* (*E. multilocularis*), is a severe parasitic zoonosis that is potentially fatal for humans. The parasite is primarily transmitted by wildlife, with red foxes acting as definitive hosts and rodents as intermediate hosts, while humans can become accidental but dead-end hosts. The aim of this study is to use EmsB typing on *E. multilocularis* isolates from human AE cases and local animals such as foxes and rodents. In this study, retrospective EmsB typing was performed on 39 samples, including 11 tissue samples from 10 patients, 18 fecal swabs from foxes, and 10 tissue samples from rodents. A dendrogram was created to determine the EmsB profiles present. The results showed that all the rodent samples were associated with the EmsB P1 profile (10/10), while the human and fox samples shared the EmsB profile P1 (5/11 humans and 8/18 foxes), a profile near P4 (2/11 humans and 3 foxes), and a profile near P8 (1/11 humans and 1/18 foxes). The study demonstrates that the same EmsB profiles circulate among humans and animals, confirming that wildlife reservoirs play a key role in transmission.

## 1. Introduction

Alveolar echinococcosis (AE) is a zoonotic disease caused by the larval stage of the tapeworm *Echinococcus multilocularis* (*E. multilocularis*). This serious parasitic infection is a major public health problem due to its high severity. Without proper treatment, the mortality rate of AE is above 90% within 10 to 15 years [1]. In Europe, the prevalence of *E. multilocularis* has increased over recent decades, and its geographic distribution has expanded. The parasite is common in the Northern Hemisphere and is endemic in many parts of Western and Central Europe. Belgium has long been considered a low-risk country for AE, but studies on fox autopsies in Wallonia have shown increasing *E. multilocularis* prevalence (between 16.9% and 27.8%) [2,3,4]. Since 1999, locally acquired human cases of AE have been identified in Belgium, with an increasing number reported in recent years. However, this increase may be underestimated due to limited awareness among healthcare professionals.

The lifecycle of *E. multilocularis* is primarily sylvatic. In Belgium, the red fox (*Vulpes vulpes*) is the main definitive host, while rodents such as the European water vole (*Arvicola amphibius*) and the common vole (*Microtus arvalis*) act as intermediate hosts. Rodents become infected by ingesting eggs released in the feces of infected definitive hosts. These eggs develop into the larval stage of the parasite within the liver of the intermediate hosts. Infected rodents are then consumed by definitive hosts, completing the parasite lifecycle. However, humans are considered accidental and dead-end hosts. Infection occurs through the ingestion of viable eggs, typically via contaminated wild fruits, vegetables, or indirectly by the soiled fur of infected definitive hosts (foxes or dogs).

In humans, AE is caused by the larvae of *E. multilocularis* (metacestodes), which develop in the liver in 99% of cases [5]. The disease behaves similarly to cancer, with metacestode vesicles invading the liver and forming a tumor-like mass. These vesicles grow by budding and spreading, leading to the destruction of healthy liver tissue. Over time, the parasite can spread to other organs, such as the lungs or brain, causing secondary lesions.

AE progresses slowly, with an incubation period of 5 to 15 years, which complicates early diagnosis [6]. Treatment is challenging, often requiring surgery to remove part of the affected liver. Surgical outcomes are classified based on resection margins as R0 (complete resection with no microscopic residual disease), R1 (microscopic residual disease), or R2 (macroscopic residual disease). Achieving an R0 resection is crucial for improving the prognosis, as incomplete resections (R1 and R2) are associated with a higher risk of recurrence. The PNM classification system (P: parasitic lesion, N: involvement of neighboring organs, M: presence of metastases) is also used to evaluate the disease stage and guide treatment decisions [7]. In cases where surgery is not possible or the disease is advanced, long-term treatment with anthelmintic drugs, such as albendazole or mebendazole, is used to slow down the growth of the metacestodes and lead to a calcification process. However, these drugs do not cure the disease, and the prognosis remains poor if diagnosed at a late stage. Early detection and treatment are essential for improving patient outcomes.

The EmsB microsatellite marker is a repetitive DNA sequence, characterized by (CA)ₙ and (GA)ₙ patterns (n indicating the number of repeats), located on chromosome 5 of *E. multilocularis*. It is highly polymorphic and has proven to be particularly effective for differentiating strains or profiles of this species, making it a mainstay in genetic studies [8,9,10,11]. Microsatellites, or simple sequence repeats (SSRs), are short tandem repeats known for their high mutation rates, which generate significant genetic variability. This property allows microsatellite markers to offer high-resolution insights into genetic differences both within and between populations, surpassing the capabilities of mitochondrial and nuclear markers [12]. These advantages are crucial for understanding genetic diversity and epidemiological dynamics. The EmsB marker has been extensively used to analyze *E. multilocularis* isolates from Central and Northern Europe, where AE is endemic [13,14,15,16]. From a European cohort of 63 AE patients, the genetic diversity of *E. multilocularis* was investigated through the analysis of the microsatellite EmsB and nine EmsB profiles (P1 to P9) were highlighted, with a numerical predominance of the profile P4, clustering patients on either side of the French, Germans and Swiss borders, including an AE lesion of a Belgium patient being characterized by a P1 profile [17]. This was confirmed by a study on a Belgian cohort (n = 16), with four profiles described amongst patients (P1, P4, P8, and P9) [18]. Its application has enabled precise tracking of infection patterns and identification of genetic variation at both broad and localized geographic scales. Despite challenges in determining the time and location of human infections due to the parasite’s long incubation period, EmsB has facilitated tracing transmission routes and the study of host-parasite interactions. Data generated through EmsB typing are centralized in the EmsB Website for the Echinococcus Typing (EWET) database, a valuable resource for comparing genetic profiles across regions [19]. This database supports research efforts and public health interventions, particularly in regions where AE is spreading.

The aim of this study is to perform EmsB typing on *E. multilocularis* isolates, including human samples from AE patients and samples from local animal hosts such as foxes and rodents. This approach follows the One Health concept, which considers the connections between human health, animal health, and the environment to better understand and control the disease.

## 2. Materials and Methods

### 2.1. Sample Collection

Between 2021 and 2023, 11 biological samples were collected from 10 patients diagnosed with AE and referred to the University Hospital of Liege (CHU de Liege, Belgium) for diagnostic confirmation or therapeutic management. Most of the patients were Belgian, except for one from Luxembourg. The samples were obtained during surgical interventions or via needle aspiration and were either fresh (n = 9) or formalin-fixed paraffin-embedded samples (FFPE) (n = 2), provided by the Pathology Department of the CHU of Liege. The majority were derived from the liver (n = 8), while the other samples originated from the lungs (n = 2) and cerebrospinal fluid obtained via lumbar puncture (n = 1). One patient had two liver samples collected during a single surgery in 2022, from segment V and the intersection of segments VII/VIII. Table 1 presents the clinical data of the AE patients included in this study.

The animal-derived specimens included 18 fecal swabs from foxes (*Vulpes vulpes*) collected in the Namur, Liege, and Luxembourg provinces and 10 liver tissue samples from rodents (muskrat—*Ondatra zibethicus*), collected in the Hainaut and Namur provinces. These samples, collected between 2021 and 2023, were provided by the Fundamental and Applied Research for Animals & Health unit (FARAH, Faculty of Veterinary Medicine, University of Liege). These samples were collected from dead animals.

An in-house PCR confirmed the presence of *E. multilocularis* DNA in all the human and animal samples.

### 2.2. DNA Extraction

DNA extraction was carried out using the Maxwell AutoMate (Promega, Madison, USA) and corresponding extraction kits, following the specific protocols and recommendations of the manufacturer for each type of sample. For the fresh samples, including tissue and cerebrospinal fluid, the Maxwell 16 LEV Tissue Kit (Promega, Madison, WI, USA) was used, with an initial one-hour treatment at 56 °C with proteinase K (Qiagen, Hilden, Germany). For the FFPE samples, a specific protocol was followed. The tissue sections, prepared with a Leica Histocore Autocut microtome (Leica Biosystems, Heidelberg, Germany), were deparaffinized using xylene and treated with proteinase K at 70 °C for two hours. DNA extraction was then performed using the Maxwell 16 FFPE Plus LEV DNA Purification Kit (Promega, Madison, WI, USA). For two fresh tissue samples, DNA extraction was done manually using the QIAamp DNA Mini Kit (Qiagen, Hilden, Germany) to achieve better DNA yield. DNA concentration was measured using a NanoDrop ND-1000 spectrophotometer (ThermoFisher, Waltham, MA, USA). All the DNA samples were stored at −20 °C before performing PCR.

### 2.3. EmsB Microsatellite Amplification and Data Analysis

EmsB typing was performed following the method described by Knapp et al. (2007) [8]. PCR amplification was carried out using two specific primers: EmsB A (5′-Fam-GTGTGGATGAGTGTGCCATC-3′), labeled with 6-carboxyfluorescein (6-FAM), and EmsB C (5′-CCACCTTCCCTACTGCAATC-3′). To verify the reliability of the results, each sample was tested in duplicate. Samples with inconsistent results or no detectable EmsB profile were excluded from further analysis. A calibrator plasmid containing four EmsB microsatellite sequences was included in each PCR run to validate the accuracy of the electrophoresis [19]. The PCR products were analyzed using capillary electrophoresis on an ABI 3500 sequencer (ThermoFisher Scientific, Waltham, MA, USA). The data were processed with GeneMapper software V5.0 (ThermoFisher Scientific, Waltham, MA, USA), which determined the fragment sizes in base pairs (bp) and fluorescence intensities of the peaks. These peaks, typically ranging from 209 to 241 bp, represented alleles, with their height corresponding to the number of microsatellite copies in the parasite DNA [9]. Peaks with fluorescence intensity below 10% of the highest peak in a run were considered artifacts and excluded from the analysis. To account for differences in DNA concentration, peak intensities were normalized by dividing each value by the total intensity of all the peaks for a given sample [19].

To ensure the reliability of the data, the profiles were validated using specific criteria, including reproducibility between duplicate samples and the overall quality of the electropherograms, particularly the number and distribution of peaks. Samples that did not meet these criteria were excluded from genetic analysis. To study genetic relationships among samples, a hierarchical clustering approach was used. Euclidean distances among samples were calculated, and the unweighted pair group method with arithmetic mean (UPGMA) was applied to construct a dendrogram using R statistical software version 3.6.1 (R Development Core Team, 2024). A threshold of 0.1 in Euclidean distance was set, as modified by [17], to define genetic relationships and assign EmsB profiles (Pn), where n corresponds to the profile number, according to [17]. To enhance the quality of analysis, additional reference profiles were included in the dendrogram. These profiles consisted of 80 samples from the EWET database, and 18 Belgian profiles reported by Sacheli et al. (2023) [18]. The EWET database profiles included samples from the United States (n = 3), Japan (n = 1), Austria (n = 1), Germany (n = 3), Switzerland (n = 22), France (n = 46), and Belgium (n = 1). These reference profiles were previously characterized by Knapp et al. (2007, 2020) [8,17]. Samples with a Euclidean distance below 0.1 were considered to belong to the same EmsB profile.

### 2.4. Statistical Analysis

We performed a correlation analysis using a Spearman test (R Studio software, version 3.6.1) to assess the similarity between samples BE_EmsB_H_03 and BE_EmsB_H_04. The correlation between the EmsB microsatellite genetic profiles and the place of residence in Belgium or Luxembourg was assessed using Fisher’s exact test. The association between EmsB profiles and clinical features was evaluated using ANOVA or Chi-square tests, depending on the variable type. A significance level of 5% (*p* < 0.05) was applied. The diversity of the EmsB profiles was assessed using the Simpson index (D). The evenness index (E) was then calculated as E = 1 − D, reflecting both the number and relative abundance of the profiles within the population. Index D quantifies the probability that two randomly selected EmsB profiles from the population are identical. This index ranges from 0 to 1, with values closer to 1 indicating lower diversity and a higher prevalence of a particular EmsB profile. Index E assesses the distribution balance of EmsB profiles, also ranging from 0 to 1, where values approaching 1 suggest an equitable distribution of EmsB profiles within the locality. All the statistical analyses were conducted using SAS software version 9.4.

## 3. Results

Regarding the human samples, a total of 11 samples were analyzed, collected from nine AE Belgian and one AE Luxembourgish patients. The sex ratio was 4:6 (males: 40%). The mean age at diagnosis was 56.6 years, with an age range of 25 to 77 years. At the time of diagnosis, 60% of the patients (6/10) were symptomatic. Serological testing (Em2-Em18 IgG ELISA, Bordier Affinity Products, Crissier, Suisse) was positive in all the cases (10/10). Hepatic involvement was observed in all the patients, with 20% (2/10) also showing extrahepatic lesions. Radical surgical resection (R0) was performed in 30% of the cases. Statistical analysis using ANOVA and Chi-square tests found no significant associations (*p* > 0.05 for all variables) between EmsB profiles and clinical characteristics (sex, age at the time of diagnosis, extrahepatic localization, symptoms, PNM classification, and R0 curative surgery). The main clinical characteristics of the patients are presented in Table 1.

**Figure 1:**
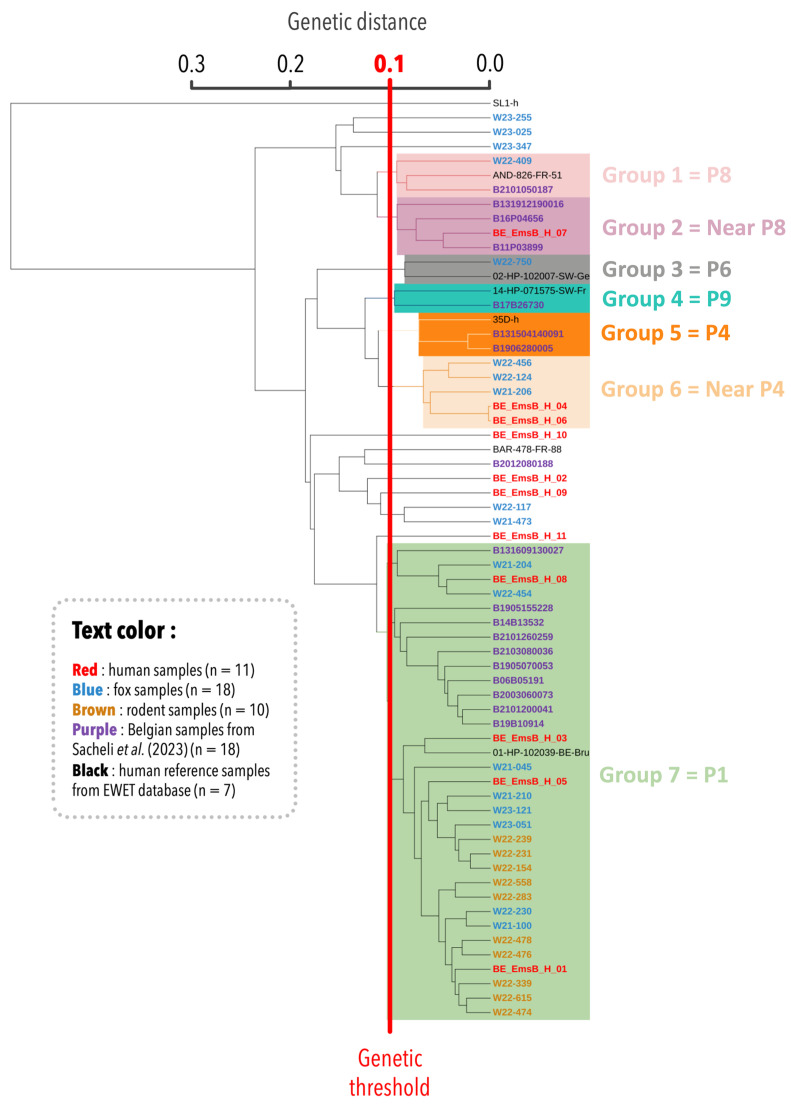
Dendrogram showing Euclidean distance classification based on EmsB microsatellite analysis. The analysis is based on 11 human samples (in red), 18 fox samples (in blue), 10 rodent samples (in brown), 18 Belgian patient samples from [18] (in purple), and 7 human reference samples from the EWET database (in black). The red line indicates the 0.1 Euclidean distance threshold.

Genetic relationships among the samples analyzed in this study were assessed. This analysis included a total of 56 samples: 39 from this study, 18 Belgian human samples from a previous study published by [18], and seven human reference samples from the EWET database. This expanded dataset provided crucial insight into the genetic diversity and geographic distribution of *E. multilocularis*. The EWET samples were chosen to represent EmsB profiles P1, P4, P6, P7, P8, and P9, along with the Alaskan *E. multilocularis* outgroup, ensuring the inclusion of diverse genetic profiles. The resulting dendrogram revealed seven main groups (Figure 1). Group 1 contained three samples: one Belgian fox sample, one Belgian human sample, and one reference sample from the EWET database identified as the EmsB P8 profile. Group 2 formed a distinct clade but remained closely related to the P8 profile. At this stage, it has been designated as a “Near P8” profile due to its proximity to the P8 profile. Group 3 included the reference sample for the P6 profile along with a Belgian fox sample clustered within this profile. Group 4 contained two samples, including one from the EWET database (EmsB P9 profile) and the B17B26730 sample from [18]. The latter was previously near but not definitively within the P9 profile, clustered with the P9 profile in this analysis, further supporting the genetic similarity of these samples. Group 5 consisted of three samples from previous studies, all forming the EmsB P4 profile. Group 6 included two Belgian human samples from this study and three fox samples. This group has not yet been assigned a definitive classification. However, given its proximity to the P4 profile, it has been preliminarily designated as “Near P4”. Finally, group 7, the largest and most significant group, included most of the analyzed samples (33/56) from this study. This grouping highlights the close genetic relationship between Belgian human (n = 4) and animal samples (rodents: n = 10, foxes: n = 8). Regarding the Belgian human samples from a previous study, 10 out of 18 were clustered within group 7, which was associated with the P1 profile. A sample identified by [17] as P1 also fell within this group. Five fox samples and three human samples did not cluster within any defined group.

**Figure 2:**
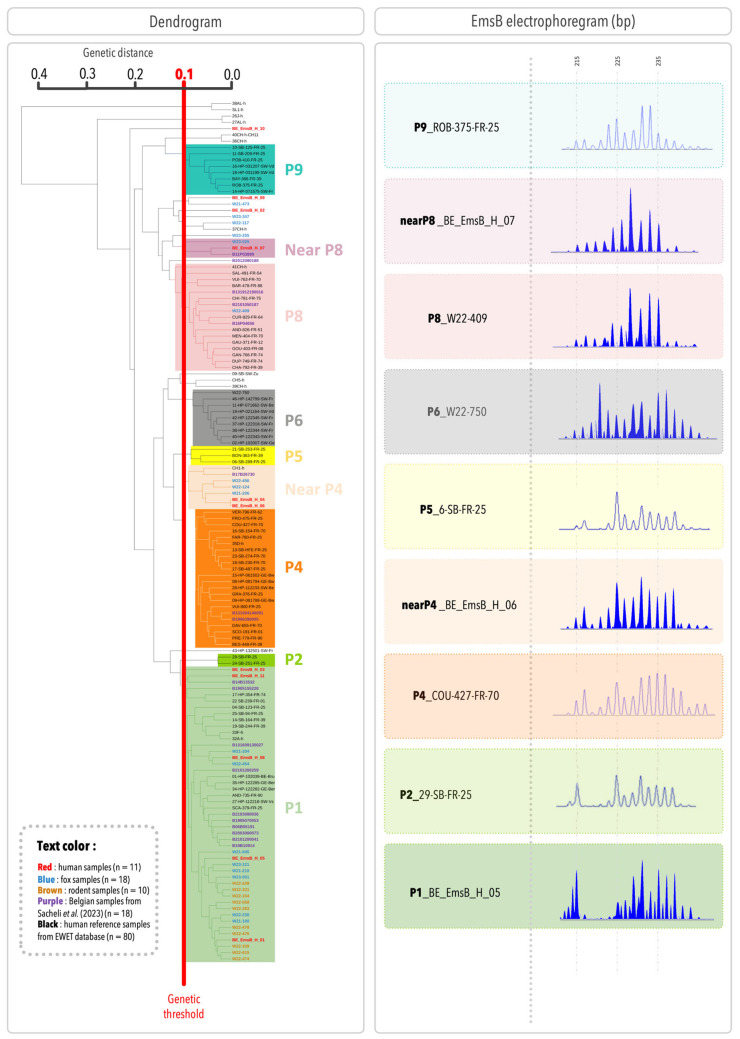
Dendrogram showing the Euclidean distance classification based on EmsB microsatellite typing, with EmsB profiles delineated. The analysis includes 11 Belgian human samples (in red), 18 fox samples (in blue), 10 rodent samples (in brown), 18 Belgian patient samples from [18] (in purple), and 80 human reference samples from the EWET database (in black). The red line indicates the 0.1 Euclidean distance threshold. On the right side of the figure, a representative EmsB electropherogram is shown for each profile.

In Figure 2, the dendrogram includes the samples from the present study, Belgian samples from [17], and 80 European human samples, resulting in a total of 137 samples. Adding more samples improves the accuracy and coverage of the genetic analysis by representing a broader range of genetic diversity. To be consistent with previous studies, the profile numbering system from the previous studies was used [17]. Two human samples (BE_EmsB_H_03 and BE_EmsB_H_11) were closely related to the P1 profile, but with a Euclidean distance slightly above 0.1, could not be classified within this clade. To further investigate, an individual search was conducted using the entire EWET database (1450 samples as of 19 February 2025). The analysis confirmed that these samples were most closely related to the reference samples of the P1 profile. Therefore, they were considered part of the P1 profile. Based on this classification, the patient samples in the present study were grouped into three main profiles: EmsB P1, “Near P4”, and “Near P8”. These profiles correspond to group 7, group 6, and group 2 in Figure 1, respectively. They were also identified in definitive hosts, with 44% of foxes grouped in the EmsB P1 profile (8/18), 17% in the “Near P4” profile (3/18), and 6% in the “Near P8” profile (1/18). Additionally, one fox sample was clustered within the P6 profile, and another within the P8 profile. All the intermediate hosts (rodents) were classified into the P1 profile (10/10). Three human samples and four fox samples did not cluster within a well-defined clade. Among them, BE_EmsB_H_09, identified as an outgroup, has a Euclidean distance below 0.1 with W21-473 but does not form a distinct clade. Due to the low number of samples, no classification can be assigned at this stage. Two samples, taken from different liver segments of the same patient (segment V and the junction of segments VII and VIII), showed different EmsB profiles; one was clustered with the P1 specimens and the other with the “Near P4” samples, suggesting genetic diversity even within the same host. From a statistical perspective, the Spearman test shows a significant correlation (correlation factor = 0.79, *p* = 0.00014), indicating a clear association between the two hepatic samples from the same patient. However, the Euclidean distance (0.15) exceeds the established threshold (0.10), suggesting that while the samples share a similar trend, they remain distinct. Moreover, the spectra of these two samples are not identical, further supporting the idea that they have close but different profiles. The Belgian samples from a previous study were classified into the same profiles as initially described, except for two. Sample B131609130027, previously considered an outgroup but close to the EmsB P1 profile, is now classified in the EmsB P1 profile. Similarly, sample B17B26730, initially an outgroup but close to the EmsB P9 profile, is now in the EmsB Near P4 profile. This shows how dendrogram results can change as more samples are added, giving a clearer view of genetic relationships. A representative EmsB electropherogram for each profile is shown in Figure 2.

**Figure 3:**
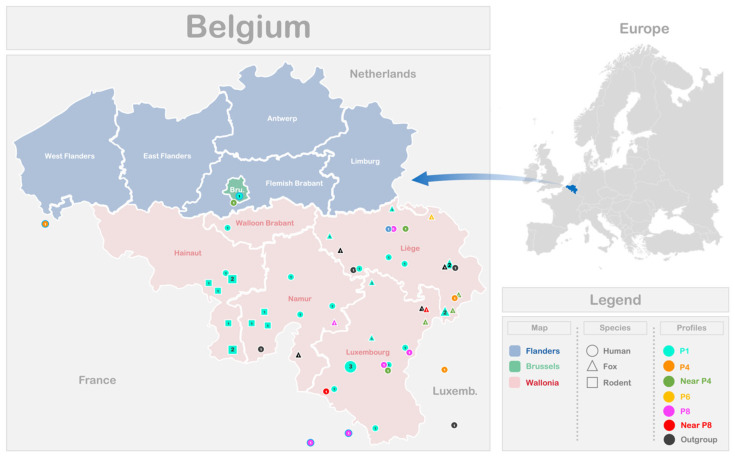
Geographical distribution of *Echinococcus multilocularis* EmsB profiles in Belgium, including samples from AE patients and animal hosts in this study. Data from Belgian patients [18] are also included. Four samples from [17] were added and are outlined in blue. Shapes and colors represent different hosts and EmsB profiles, respectively.

The distribution of the different EmsB profiles identified among the 39 samples analyzed in this study, originating from various Belgian and Luxembourg regions, is illustrated in Figure 3. Additionally, the profiles reported in a previous Belgian study [18] are included. To enhance visualization of circulating profiles near the border, data from three French samples collected at the Franco–Belgian border and one Belgian sample from the study by Knapp and co-workers [17] were also included. The P1 profile, regardless of the host species, was present across all the Belgian provinces examined in this study. However, no significant association was found between the EmsB profiles and the patient’s place of residence or the location of animal discovery, as indicated by Fisher’s exact test (*p*-value = 0.19 for human samples; *p*-value = 0.57 for animal samples). The lack of a significant relationship may be due to the limited sample size or low variability in the dataset. To assess the diversity and uniformity of the EmsB profiles across localities, Simpson’s index (D) and the evenness index (E) were calculated. For both human and animal samples, Simpson’s index (D) values are low, indicating relatively high diversity of EmsB profiles (humans: D = 0.27; animals: D = 0.38). The evenness index (E) for human samples (E = 0.72) suggests a relatively equitable distribution of profiles, while for animal samples (E = 0.61), the index indicates moderate equitability, with some profiles being more frequent than others, although diversity remains present. These statistical analyses confirm that no significant correlation exists between the EmsB profiles and geographic location. For the animal samples, the discovery locations do not cover all the provinces of Wallonia. The rodent samples are limited to the Hainaut and Namur provinces, while the fox samples originate from the Namur, Liege, and Luxembourg provinces.

## 4. Discussion

The EmsB microsatellite marker has become an invaluable tool for understanding the genetic diversity and population structure of *E. multilocularis*. This typing method provides detailed insights into the EmsB profiles of the parasite, allowing researchers to trace transmission pathways, study host–parasite interactions, and monitor changes in parasite populations over time. Its ability to differentiate among *E. multilocularis* isolates has proven essential for unraveling the epidemiology of AE and assessing the role of wildlife and humans in the transmission cycle.

The first study characterizing the EmsB marker was conducted by Bart et al. in 2006 [9]. This study analyzed 35 *E. multilocularis* isolates from different parasitic stages (adult and metacestode) and various hosts (foxes, rodents, and humans). The results demonstrated that the EmsB marker is the most suitable marker for studying the genetic diversity of *E. multilocularis*, as it possesses three key properties of an effective genetic marker: high sensitivity, reproducibility, and strong discriminatory power. These characteristics make the EmsB marker particularly valuable for investigating the genetic diversity and population structure of *E. multilocularis*.

Since then, EmsB typing has been widely applied to *E. multilocularis* isolates from both animal hosts and humans across Europe and beyond. By adopting a One Health approach, this typing method allows researchers to study the interconnections between key hosts, as well as their interactions with the environment. Many of these studies have been conducted with the involvement of the French National Reference Center for Echinococcoses (University Hospital of Besançon, France), which has contributed to the development and characterization of the EmsB marker and played a key role in establishing methodological guidelines for EmsB analysis applied to AE. A detailed guide outlining standardized protocols has been made publicly available (https://ewet-db.univ-fcomte.fr/public/EmsB_analysis_guidelines.pdf) (accessed on 17 May 2025) to assist researchers in implementing this typing method. To centralize EmsB typing data, the EWET database was created in 2017. At that time, this open-access resource compiled genetic data from 1166 *E. multilocularis* isolates collected from wild, domestic, and human hosts across various European, Asian, and North American countries [19]. By integrating data from multiple endemic regions, the database facilitates comparative analyses between endemic and peripheral areas.

EmsB typing has been successfully applied in several countries, including France, Switzerland, Belgium, Germany, Poland, Denmark, Sweden, China, Japan, and North America, among others [13,14,17,20,21]. The increasing amount of data generated through this method allows for comparisons between countries, helping to identify potential similarities and better understand the distribution and variability of the parasite. In Belgium, the first large-scale national study was conducted by Sacheli et al. in 2023 [18]. Before this research, EmsB microsatellite typing in Belgian human and animal populations had been largely underexplored. Indeed, only one human isolate and 43 red fox isolates had been analyzed in multicenter studies [13,17]. The human sample had an EmsB P1 profile and was isolated in a patient from Brussels in 2010 (sample 01-HP-102039-BE-Bru) [17].

The first large-scale Belgian study analyzed 18 human samples, with 14 successfully classified into three distinct EmsB profiles: P1, P4, and P8. The P1 profile was found to be the most common, which is confirmed by our data. Building on this work, the present study included human samples collected from 2021 to 2023 and introduced animal host samples (from foxes and rodents). The analysis achieved three main goals: (i) to determine if the EmsB profiles found in human cases align with those reported earlier, (ii) to investigate whether these profiles are also present in animal hosts, and (iii) to examine the geographical distribution of EmsB profiles across different provinces in Belgium. Additionally, this study assessed whether the EmsB profiles identified in Belgium are unique by comparing them with profiles documented globally. The data included in our study were compared with previously typed data to define EmsB profiles. The inclusion of additional data improved the classification and enhanced the accuracy of profile groupings. Including a wider range of samples improved the accuracy of Euclidean distance calculations, enhancing the ability to differentiate and interpret genetic relationships.

The study reveals that Belgian samples from both animals and humans predominantly share three EmsB profiles: P1, Near P4, and Near P8 profiles. This suggests a close relationship between the parasite and these different hosts. It also supports the hypothesis of endemicity of the parasite in Belgium and indicates that the infection likely occurs within the country. At this stage, Belgium appears to have one persistent EmsB profile (P1), as the first human cases identified with EmsB P1 date back to 2006. This profile has remained stable over time and has also been identified in other European countries such as France, Switzerland, and Germany [19]. In this latter study, of 66 human samples from patients with AE who underwent surgery between 1981 and 2019, nine EmsB profiles were identified. The dominant profile in this study was P4, detected in 29% of the samples and identified in France, Germany, and Switzerland. The P1 profile was also found in French, Belgian, Swiss, and German samples. This suggests that the P1 and P4 profiles are common and widely distributed in Europe. As for the P8 profile, it was found in 14% of the samples and was exclusively identified in patients from France. However, in this study, two samples from the Franco–Belgian border both exhibited the EmsB profile P8, which was also found among Belgian human and fox samples. This could indicate cross-border transmission of the parasite. The similarity of the profiles found in Belgium with those in other European endemic areas supports the idea of connected endemic zones. The P1, P4, and P8 profiles have been described as widely distributed, whereas the P6 profile, for example, is considered a local profile, predominantly found in Switzerland [19]. The study by Umhang et al. (2021) provided evidence of the shared distribution in animal hosts of EmsB profiles across multiple European countries [13]. This study analyzed 785 *E. multilocularis* isolates, mainly from adult worms isolated from red fox intestines (*Vulpes vulpes*) and collected from four Western European countries, nine Eastern European countries, as well as Armenia and the Asian regions of Russia and Turkey. The findings revealed a close genetic relationship among *E. multilocularis* populations from Germany, Belgium, and Luxembourg. Specifically, in Western Europe, four countries (Germany, Belgium, Luxembourg, and the Netherlands) shared six common EmsB profiles, which accounted for 70% of the samples analyzed. The shared profiles could be attributed to foxes or domestic dogs moving across neighboring countries, or to patients becoming infected while abroad. Unfortunately, comparing the EmsB profiles with those in the present study is difficult because the profiles’ numbering systems are different (WEn numbering, where “n” refers to a clade number, while the present study uses Pn numbering). These findings also emphasized the need for continued testing of human and animal samples to determine whether the same trend persists or new profiles emerge.

Among the EmsB profiles shared between humans and foxes, the Near P4 (two human and three fox samples) and Near P8 (one human and one fox sample) profiles were identified. These profiles form a distinct clade while remaining genetically close to the P4 and P8 profiles, respectively. To facilitate future studies, these profiles were considered as distinct profiles. In fact, this approach allows future studies to determine whether these samples eventually cluster within the known P4 and P8 profiles or if additional samples will group within the Near P4 and Near P8 profiles, thereby confirming their distinct nature. Only further data can clarify this classification. Additionally, three human samples and four fox samples did not cluster into any defined clade. Despite an extensive search across the entire EWET database, no known profile could be assigned to them. This suggests that the EWET database is not yet exhaustive and that these samples possess unique EmsB profiles that have not been identified so far. However, even though these samples do not form a well-defined clade, sample BE_EmsB_H_09 has an Euclidean distance below 0.1 with W21-473. This further supports the observation of genetic proximity between *E. multilocularis* in human and animal hosts.

All the rodent samples (n = 10) included in this study were clustered within the EmsB clade P1. This consistent clustering may reflect a limited diversity of *E. multilocularis* strains circulating in intermediate hosts in the studied areas of Belgium. One possible explanation is the territorial behavior of rodents, which results in geographically restricted and poorly mobile populations. This limited dispersal could reduce their exposure to diverse parasite genotypes, leading to a predominance of a single clade. However, due to the small sample size, further investigations are needed to confirm this trend and assess its epidemiological implications.

No significant link was found between EmsB profiles and clinical characteristics of patients (sex, age at the time of diagnosis, extrahepatic localization, symptoms, PNM classification, and R0 curative surgery). However, an observation was made involving two samples from the same patient, taken from different liver segments (segment V and the intersection of segments VII and VIII) during a single surgical resection in 2022 (BE_EmsB_H_03 and BE_EmsB_H_04). These samples were grouped into two separate EmsB profiles (P1 and Near P4) based on Euclidean distance calculations. This suggests that the patient may have been infected by two different *E. multilocularis* strains. This could happen through repeated exposure to parasite eggs from various sources, such as contact with infected animals like foxes, or through contaminated food or water. Eggs from different infected animals can coexist in the environment, increasing the risk of being infected by multiple strains. Another possibility is sequential infections, where a second infection occurs before the first is detected or treated, due to the long incubation period of *E. multilocularis*. Strains from different infections might also settle in different liver segments if infections happen at different times. The identification of two EmsB profiles within a single human host may influence future diagnostic and therapeutic approaches by highlighting the possibility of mixed infections. This could affect genotype detection accuracy and potentially treatment response. Epidemiologically, it highlights greater parasite diversity within hosts, which could affect transmission dynamics and control strategies. Co-infection with multiple *E. multilocularis* strains, well described in foxes, highlights the complex ways the parasite spreads and interacts with hosts and the environment [22]. More molecular research is needed to better understand this phenomenon. This is the first observation of two different EmsB profiles in the same patient using EmsB typing. The previous studies have shown that multiple samples collected from the same patient present identical EmsB profiles [17,18].

Most of the samples analyzed in this study come from the Wallonia region (southern part of Belgium), except for two human cases: one from the Flemish Brabant province and another from Luxembourg, both bordering Wallonia, which appears to be more endemic for *E. multilocularis* than Flanders, as demonstrated by several studies [2,4,23,24,25]. The higher endemicity of *E. multilocularis* in Wallonia compared to Flanders results from multiple ecological factors. The geographical characteristics in Wallonia, including higher altitude (altitude ranging from 400 to 700 m in altitude in Wallonia compared to 0 to 100 m in Flanders), extensive forested areas, and rural landscapes, provide optimal conditions for the parasite’s lifecycle [23,25]. Wallonia hosts a high density of intermediate hosts for *E. multilocularis*, particularly *Arvicola amphibius* and *Microtus arvalis*, which are crucial to the parasite’s lifecycle and present elevated infection rates [2]. While muskrats (*Ondatra sp.*) are present across Belgium, they have notably higher infection rates with *E. multilocularis* in Wallonia than in Flanders [2,24,26]. Moreover, in Flanders, foxes primarily feed on brown rats (*Rattus norvegicus*), which are less suitable hosts for *E. multilocularis* [25]. Intensive agriculture and urbanization reduce *Arvicolidae* habitats, contributing to lower fox parasite prevalence rates [25]. Climatic conditions with higher rainfall and cooler temperatures further support the survival of *E. multilocularis* eggs, while the earlier expansion of red fox populations in the 2000s, after the European rabies outbreak and pressure on foxes, has facilitated the establishment and spread of *E. multilocularis* [2,23]. These combined factors create a more favorable ecosystem for *E. multilocularis* in Wallonia, explaining its significantly higher prevalence compared to Flanders. Currently, although patients or animals from the same area had identical or similar profiles, no specific profile is linked to a particular province. The EmsB profiles identified in the Belgian samples are evenly distributed across Wallonia in both human and animal hosts, except for rodents. The study is limited by the small number of rodent samples from the Hainaut and Namur provinces, which prevents determining EmsB profiles in rodents from other Walloon provinces where human and fox EmsB profiles have been identified. This geographical limitation highlights the need for future research to expand sampling across a broader range of provinces. The EmsB P4 profile was identified in two human samples in the study reported by [18]. One sample originated from Luxembourg, while the other was from the province of Liege, located at the border with Luxembourg and Germany. This distribution suggests that the EmsB P4 profile might not be endemic to Belgium. A plausible hypothesis is that these patients could have been infected in neighboring countries rather than in Belgium itself. The proximity of these cases to national borders and the absence of the P4 profile in other Belgian samples support this theory, highlighting the potential for cross-border transmission of *E. multilocularis* strains. Another possible hypothesis is the migration of foxes from Luxembourg or Germany into Belgium, carrying the EmsB P4 profile. To investigate this, the study would need to be expanded to include a larger sample of foxes from border regions to determine whether this hypothesis can be supported. Four samples identified as the EmsB Near P4 profile (one human and three fox samples) are from the provinces of Luxembourg and Liège, near the borders of Luxembourg and Germany, where the P4 profile was previously reported. This Near P4 profile is genetically similar to the P4 profile but exhibits slight variations, which may suggest ongoing local diversification or evolutionary changes over time.

One key limitation of EmsB typing is the use of a Euclidean distance threshold of <0.1 to define genetically similar samples. This threshold may not fully reflect genetic diversity, as samples with slightly higher distances could still be closely related. This limitation should be considered when interpreting genetic relationships based on EmsB typing. As an alternative, Bayesian clustering methods could provide a more robust approach by inferring population structure without relying on an arbitrary distance cutoff. This approach can better reveal the underlying population structure and genetic relationships.

## 5. Conclusions

This study shows that the same EmsB profiles are shared between human and animal hosts, highlighting the key role of wildlife reservoirs in the transmission of *E. multilocularis*. Comparisons with reference specimens from the EWET database and previous studies further support these findings. Belgium shares EmsB profiles with other European countries, emphasizing the interconnected nature of endemic regions. Moreover, some outgroup samples have been identified, indicating the possible presence of unique profiles in Belgium. The detection of multiple profiles within a single patient suggests possible infection by different parasite eggs, which needs further investigation into the underlying mechanisms. No geographic correlation was observed between EmsB profiles and geographic location. To confirm and expand these findings, it is crucial to increase the sampling of foxes and rodents across Wallonia, including regions with varying environmental and ecological conditions. This will help ensure that the observed patterns are representative and comprehensive. This research significantly advances our understanding of *E. multilocularis* genetic diversity in Belgium and provides a critical foundation for future epidemiological and molecular studies. This study underscores the need for public health strategies within a One Health framework. These should include the identification of high-risk areas, epidemiological monitoring, improved awareness campaigns (particularly in endemic zones), and optimization of screening programs to better prevent and control AE.

## Figures and Tables

**Table 1:** Main clinical characteristics of patients with alveolar echinococcosis included in this study.

ID	Sex	Age at the Time of Diagnosis	Year of Surgery	Organ Sample	Extrahepatic Localization (Yes/No)	Type of Biological Material (FFPE or Fresh)	Symptoms (Yes/No)	Serology	PNM Classification	R0 Curative Surgery (Yes/No)	Postal Code	Country	EmsB Profile
BE_EmsB_H_01	F	64	2021	Liver	No	Fresh	Yes	Positive	P1N0M0	Yes	6852	Belgium	P1
BE_EmsB_H_02	M	77	2021	Liver	No	FFPE	No	Positive	P1N0MO	No (R2)	5886	Luxembourg	OUT
BE_EmsB_H_03 *	F	51	2022	Liver (segment VII–VIII)	No	Fresh	Yes	Positive	P1N0M0	Yes	6640	Belgium	P1
BE_EmsB_H_04 *			2022	Liver (segment V)		Fresh							Near P4
BE_EmsB_H_05	F	25	2022	Lung	Yes	Fresh	Yes	Positive	P4N1M1	No	6060	Belgium	P1
BE_EmsB_H_06	F	42	2022	Liver	No	Fresh	No	Positive	P3N0M0	No (R1)	1640	Belgium	Near P4
BE_EmsB_H_07	F	69	2021	Liver	No	Fresh	Yes	Positive	P1N0M0	No (R1)	6831	Belgium	Near P8
BE_EmsB_H_08	M	69	2022	Liver	No	Fresh	Yes	Positive	P1N0M0	No (R1)	6600	Belgium	P1
BE_EmsB_H_09	M	63	2023	Liver	No	FFPE	No	Positive	P1N0M0	Yes	4750	Belgium	OUT
BE_EmsB_H_10	M	33	2023	Lung	Yes	Fresh	Yes	Positive	P2N0M1	No (R1)	4560	Belgium	OUT
BE_EmsB_H_11	F	73	2023	Cerebrospinal fluid	No	Fresh	No	Positive	P4N0M0	No	4560	Belgium	P1

ID = Identification; M = male; F = female; FFPE = Formalin-Fixed Paraffin-Embedded tissue; Fresh = Unfixed samples in original condition; R1 = Microscopically incomplete resection; R2 = Macroscopically incomplete resection; PNM classification: P = parasitic mass in the liver, N = involvement of neighboring organs, and M = metastasis. * Samples from the same patient.

## Data Availability

The original contributions presented in this study are included in the article. Further inquiries can be directed to the corresponding author.

## Abbreviations

The following abbreviations are used in this manuscript:

AE	Alveolar echinococcosis
FFPE	Formalin-Fixed Paraffin-Embedded
Bp	Base pairs
